# Thyroid Gland Disorders and Physical Activity: Can They Affect Each Other?

**DOI:** 10.7759/cureus.81489

**Published:** 2025-03-31

**Authors:** Maria Gavriilidou, Angeliki Chorti, Aggeliki Psomiadou, Eirini Koidou, Maria Papaioannou, Theodosios Papavramidis

**Affiliations:** 1 Faculty of Medicine, School of Health Science, Aristotle University of Thessaloniki, Thessaloniki, GRC; 2 1st Propedeutic Department of Surgery, AHEPA University Hospital, Aristotle University of Thessaloniki, Thessaloniki, GRC; 3 1st Propedeutic Surgical Department, AHEPA University Hospital, Aristotle University of Thessaloniki, Thessaloniki, GRC; 4 Physical Education School, Aristotle University of Thessaloniki, Thessaloniki, GRC; 5 Laboratory of Biological Chemistry, Faculty of Medicine, School of Health Sciences, Aristotle University of Thessaloniki, Thessaloniki, GRC

**Keywords:** exercise, physical activity, thyroid disease, thyroidectomy, thyroid hormone disorders

## Abstract

Physical activity (PA) plays a crucial role in promoting emotional well-being, quality of life, and social interaction. Thyroid dysfunction can significantly affect the ability to engage in PA. This review aims to explore the relationship between thyroid disorders and PA. A comprehensive review of the international literature was conducted, including 24 scientific studies that investigated the effects of thyroid dysfunction on exercise in both human and animal models, with and without thyroidectomy. The findings indicate that thyroid disorders are associated with a variety of conditions, including cardiovascular, metabolic, neuromuscular, musculoskeletal, and psychiatric disorders. Additionally, PA has been identified as a beneficial intervention for the management of these conditions. Further studies are needed to determine the most appropriate types and intensities of PA for people before and after thyroidectomy.

## Introduction and background

According to the World Health Organization (WHO), in 2024, physical activity (PA) is any type of movement that requires energy and involves the contraction of skeletal muscles. It is fundamental to distinguish between the terms *physical activity* and *exercise*, as the latter is a subset of the former that is planned, structured, and repetitive to enhance or sustain one or more aspects of physical fitness [1].

Insufficient PA is a significant cause of mortality worldwide and is considered a critical risk factor for non-communicable diseases (NCDs), including cardiovascular disease, cancer, and diabetes. PA plays a vital role in promoting health and preventing NCDs [2]. Unfortunately, globally, one in four adults do not engage in sufficient PA, and over 80% of adolescents worldwide exhibit inadequate levels of PA. To combat this issue, policies aimed at addressing insufficient PA have been implemented by WHO [1].

Thyroid gland disorders are common nowadays, either hyperthyroidism or hypothyroidism, and thus in women with a prevalence of 2% compared to men 0.2% [3]. The choice of PA for individuals with thyroid dysfunction depends on the type of exercise.

Athletes with hyperthyroidism have elevated thyroid hormone levels that raise their basal metabolic rate, rendering them more vulnerable to heat-related illnesses due to increased oxygen consumption and heat production [4,5]. Moreover, hyperthyroidism can cause heart rhythm changes, including arrhythmias like atrial flutter and atrial fibrillation [5,6]. Hyperthyroidism can lead to central muscle weakness (up to 67%) and rhabdomyolysis [5,7-9]. It also causes an overall loss of muscle mass due to muscle fiber atrophy. Hyperthyroidism affects bone architecture and leads to decreased bone mineral density and, thus, bone stress fractures [5,8-10].

Hypothyroidism also negatively affects bone architecture and physiology, increasing the risk of stress fractures, particularly among athletes who overtrain, have poor biometric measurements (BMI) and muscle weakness or rhabdomyolysis, or have coexisting endocrine, hormonal, or metabolic disorders [5,10,11]. Hypothyroidism can also cause bradycardia, decreased myocardial contractility, diastolic dysfunction, and a premature stiffening of blood vessels [5].

Patients receiving thyroid hormone as post-thyroidectomy replacement therapy may benefit from a home exercise program, which can reduce fatigue and stress, improve quality of life, and improve immune system function [12,13]. Research on thyroidectomy and participation in PA programs in humans is scant, although studies have been conducted on animals.

This review aims to give a detailed report on studies dealing with thyroid gland disorders and the impact of PA.

## Review

Methodology

In this review, the international studies on PubMed, Scopus, and Embase from 1888 until now were thoroughly reviewed. The search terms employed were “thyroid gland disorders” OR “thyroid gland disease” AND “physical activity” OR “exercise”. Studies meeting the following criteria were included: (1) qualitative analysis of intervention results, (2) a sample population consisting of patients with thyroid gland diseases, (3) a comparative study design, (4) inclusion of animal research, and (5) publication in English. Excluded articles were dissertations, books, recommendations, opinions, policies, or guidelines. Ethical approval is not required because this study is a review of the existing international literature.

**Figure 1:**
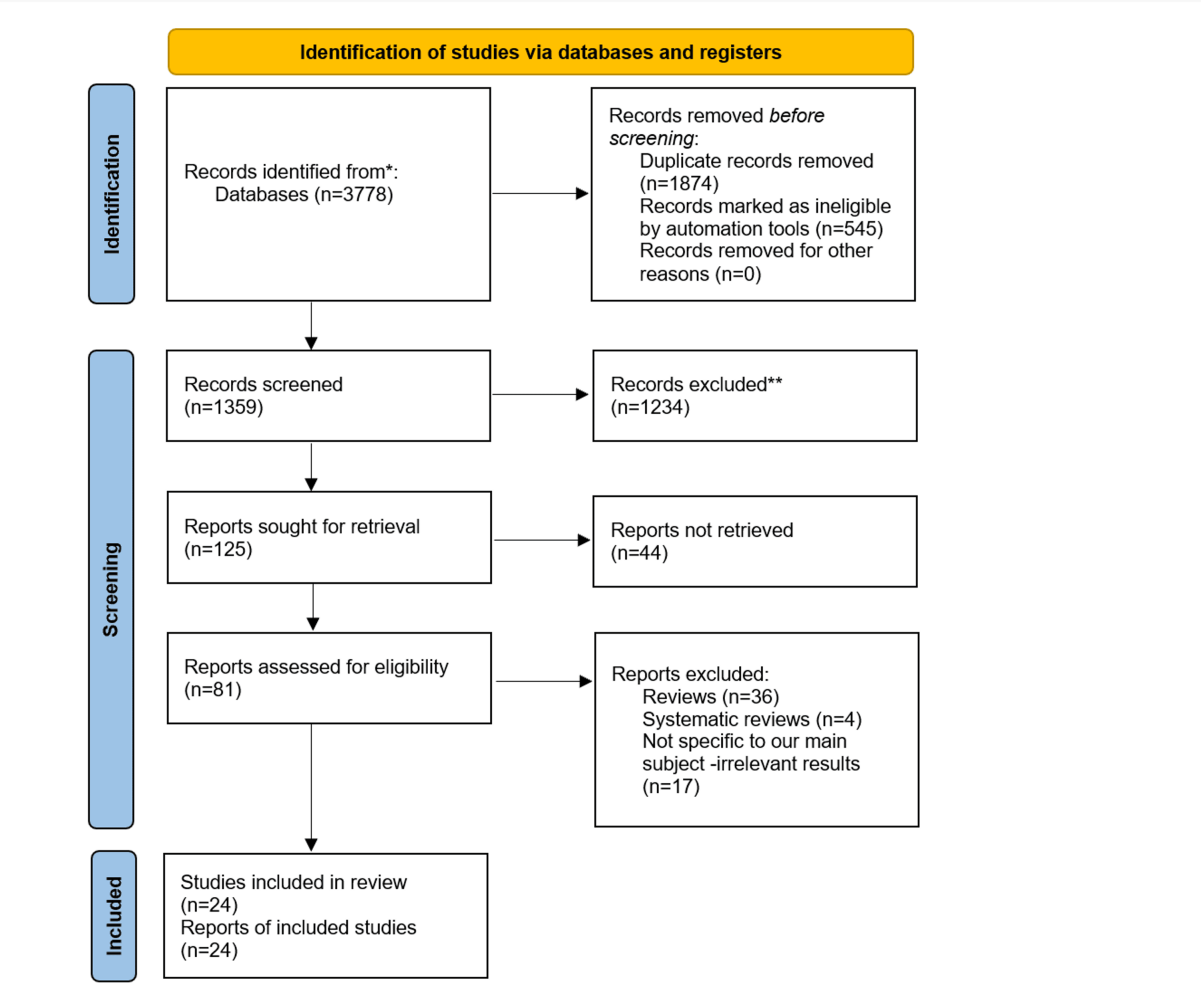
PRISMA flowchart. PRISMA, Preferred Reporting Items for Systematic Reviews and Meta-Analyses

The Preferred Reporting Items for Systematic Reviews and Meta-Analyses (PRISMA) flowchart is shown in Figure 1. From a search of three databases, 3,778 published articles were identified, as outlined in Figure 1. After removing duplicates and records flagged as ineligible by automation tools, 1,359 titles and abstracts were screened by two reviewers. A third reviewer resolved any conflicts through roundtable discussions on differing opinions. After the first screening, 1,234 articles were excluded, and all authors assessed the 125 articles according to the inclusion and exclusion criteria. Based on the criteria, 57 articles were excluded, and thus, 24 articles were included in the final review. Of the 20 articles, six were case-control studies, 11 were cohort studies, one was a retrospective analysis, and two were cross-sectional studies. Emphasizing the significance of article quality and validity within the article selection process is essential.

Review

The overall results are shown in Table 1.

**Table 1:** Studies included in the review. TSH; N/A, not available; fT4, free thyroxine; RAI, radioactive iodine therapy; FFA, free fatty acids

Author	Study type	Sample size	Exercise type	Results
Tian et al. (2024) [23]	Human study/survey analysis	5877	Physical activity	The daily physical activity of American adults is strongly associated with changes in thyroid function.
Roa Dueñas et al. (2021) [24]	Human study/Population-based cohort study	2470	Physical activity	No correlation between physical activity and thyroid hormone levels
Kocahan and Dundar (2018) [25]	Human study	20	Swimming	Elevated TSH and fT4 after exercise
Schneider et al. (2014) [26]	Human study	204	-	Hyperthyroid patients treated with surgery were less likely to gain weight postoperatively compared to RAI treatment.
Vestergaard and Mosekilde (2002) [27]	Human study	16,249	-	Hyperthyroid patients treated with thyroidectomy had lower fracture risk.
Maor et al. (2013) [28]	Human study	343	Treadmill	Hyperthyroid patients had elevated recovery and resting heart rates, and hypothyroid patients had decreased heart rates.
McAllister et al. (1995) [29]	Human study	-	All	Hyper- and hypothyroid patients exhibit exercise intolerance.
Ciloglu et al. (2006) [30]	Human study	60	Aerobic exercise	Moderate- to high-intensity exercises impact thyroid hormone circulating levels.
Schmid et al. (1982) [31]	Human study	34	Aerobic exercise	Increase in TSH after a maximal exercise bout
Ahmad et al. (2023) [32]	Human study/Randomized controlled trial	60	Aerobic and resistance training	Positive impact on TSH and T4 levels and health-related quality of life in hypothyroid patients
Lankhaar et al. (2014) [33]	Systematic review	1379	Daily sports activity	Exercise intolerance in hypothyroid patients
Guerin et al (2021) [34]	Human study/ survey analysis	580	Aerobic and resistance exercise	Hypothyroid women suffer from muscle symptoms during exercise and at rest.
Lankhaar et al. (2021) [35]	Human study	1724	Physical activity and daily sport	Hypothyroid patients, due to autoimmune thyroiditis, were less likely to achieve moderate-intensity physical activity.
Lankhaar et al. (2021) [36]	Human study	1724	Physical activity and daily sport	Hypothyroid patients in thyroid hormone replacement therapy were less likely to achieve moderate-intensity physical activity.
Tanriverdi et al. (2019) [37]	Human study	60	Physical activity	Patients with subclinical hypothyroidism are less likely to comply with exercise.
Dickson et al. (2009) [38]	Human study	62	Physical activity	Autoimmune thyroid disease patients scored better in quality of life and self-esteem survey than chronic fatigue patients.
Werneck et al. (2018) [39]	Human study/randomized controlled trial	20	Aerobic exercise	Subclinical hypothyroid women have a better quality of life after the induction of aerobic exercise in everyday life.
Park and Song (2016) [40]	Animal study/pilot study	11	Aerobic and anaerobic exercise	Exercise can help alleviate the symptoms of lethargy and reduce spatial learning ability caused by hypothyroidism.
Therminarias and Lucas (1982) [41]	Animal study	7	Exhaustive work	Thyroidectomy affects mechanisms that are more specifically involved in heat production than in muscular exercise.
Kaciuba-Uściłko et al. (1979) [42]	Animal study	N/A	Physical activity	There is an optimum level of thyroid hormones necessary to produce the typical increase in body temperature during physical activity.
Paul and Holmes (1973) [43]	Animal study	N/A	Cold exposure	The thyroid hormone does not directly control plasma FFA oxidation, but it is dependent on the metabolic rate of the tissue using it.
Teixeira et al. (2018) [44]	Animal study	40	Aerobic exercise	T3 and T4 administration could positively affect dogs intolerant to aerobic exercise after myocardial infarction.
Kasimay Çakir et al. (2017) [45]	Animal study	48	Moderate- o high-intensity exercise	A hypothyroid state could be protective in stress- and exhaustive exercise-induced oxidative damage.
Shin et al. (2013) [46]	Animal study	N/A	Treadmill	Hypothyroid rats that exercised had better outcomes compared to hypothyroid rats without exercise.

Human Studies

The first study showing that the intensity of exercise and the environmental conditions may have an impact on thyroid-stimulating hormone (TSH) secretion in the morning, but not in the evening, was published in 1981 [14]. According to scientific research, engaging in moderate to high levels of physical activity leads to positive health benefits [15]. Different forms of exercise can lead to physiological and biochemical effects in the human body [16-18]. According to Galvao and Taaffe, elastic band exercises have a positive effect on the neuromuscular system and can improve muscle strength [19]. It has also been found that physical activity can cause the formation of more free radicals by increasing metabolic processes and oxygen consumption, depending on its intensity and duration [20-22].

Physical Activity and Thyroid Function

The amount of daily physical activity of American adults is strongly related to changes in thyroid function, including thyroid hormone levels and thyroid diseases, revealed by a study with 5,877 participants in 2024 [23]. 

On the other hand, Roa Dueñas et al., in a cohort study of 2,470 patients, found no association between the endogenous thyroid hormone level and total physical activity [24]. Studies have shown that different types of exercise can positively impact the health of swimmers by affecting their lipid profiles and TSH levels. After exercising, TSH and fT4 values significantly increase compared to pre-exercise levels, as noted by Kocahan and Dundar in 2018 [25]. In concordance with aforementioned studies, in 2014, Schneider et al., in a study of 204 hyperthyroid patients, reported that hyperthyroid patients who undergo thyroidectomy are less likely to become overweight or obese than those initially treated with radioactive iodine. Furthermore, prolonged use of anti-thyroid medication may increase the risk of developing hypothyroidism and experiencing weight gain [26].

Thyroid function and musculoskeletal system

Thyroid function has an impact on the musculoskeletal system, as thyroid diseases can increase the risk of fractures. A study conducted by Vestergaard and Mosekilde in 2002 examined the incidence of fractures in patients with hyperthyroidism and hypothyroidism. The findings of the study indicated that patients diagnosed with hyperthyroidism experienced an increased risk of fractures only at the time of diagnosis. However, this risk diminished after the diagnosis and when the patient reached a normal context. The treatment of hyperthyroidism with thyroidectomy was associated with a decrease in the risk of fracture after diagnosis [27]. Surgical treatment for hyperthyroidism may lower fracture risk, unlike radioactive iodine treatment, which is associated with an increased risk of fractures [5].

Thyroid function and cardiovascular system

Thyroid dysfunction is already known to affect cardiac rate and motility. Differences in heart rate during and after exercise were observed in a study by Maor et al. in patients with hyper- and hypothyroidism. Hyperthyroid patients exert higher heart resting and recovery rates, whereas hypothyroid patients had lower values [28].

Exercise on post-thyroidectomy patients

As far as post-thyroidectomy patients are concerned, Kim et al. performed a study to evaluate the efficacy of a home exercise program for patients undergoing thyroid hormone therapy after thyroidectomy for thyroid cancer. The researchers found that the home exercise program was effective in reducing fatigue and stress, improving quality of life, and enhancing autoimmune function in patients undergoing post-thyroidectomy recovery therapy. The study highlights the potential benefits of home exercise programs as a complementary therapy for the management of post-thyroidectomy symptoms [12].

Thyroid dysfunction and exercise intolerance

The effects of hyperthyroidism on exercise tolerance were analyzed in a study carried out by McAllister et al. [29]. The researchers concluded that hyperthyroidism reduces endurance during exercise primarily through changes in energy metabolism. Moreover, the hyperthyroid state enhances cardiac and vascular functions, leading to skeletal muscle hyperexcitability during exercise. This condition is also associated with faster rates of glycogen depletion during submaximal exercise, while the biochemical changes induced by hyperthyroidism promote greater reliance on carbohydrate substrate, leading to greater glycolytic flux in muscle. Furthermore, hyperthermia, a well-known symptom of hyperthyroidism, may also contribute to exercise intolerance, and the core temperature during exercise is higher in the hyperthyroid state [29].

On the other hand, Ciloglu et al. conducted a study in 2006 to determine the impact of exercise intensity on thyroid hormones. The findings revealed that exercise performed at the anaerobic threshold (70% of maximum heart rate, lactate level 4.59 ± 1.75 mmol/L) resulted in significant changes in the levels of various hormones. Specifically, while the levels of T4, fT4, and TSH continued to rise, the levels of T3 and fT3 began to decline at 90% of the maximum heart rate. In conclusion, the study established that maximal aerobic exercise significantly affects the circulating levels of thyroid hormones. The research revealed an increase in TSH values at moderate-intensity exercise (70% maximum heart rate) and high-intensity exercise levels (90% maximum heart rate) when compared to the hormone levels at low-intensity exercise (45% maximum heart rate). Additionally, the levels of T3 and fT3 showed an increase followed by a decrease at moderate and high-intensity exercise. In opposition, fT4 and T4 values increased at moderate intensity with a continuous rise at high intensity levels [30]. These results are consistent with the Schmid et al. study, which showed that TSH continues to increase up to 15 minutes after a maximal exercise bout, with unchanged or slightly decreased T3, fT3, and fT4 [31]. Ahmad et al. proved that aerobic and resistance training can have a positive effect on T4 levels and physical health-related quality of life in hypothyroid patients, and thus the combined training can exert greater improvement in TSH [32]. However, a population-based cohort study by Roa Dueñas et al. showed no correlation between thyroid hormone levels and physical activity [24].

The impact of overt and subclinical hypothyroidism on exercise tolerance has also been examined. Untreated hypothyroid patients experience exercise intolerance due to factors negatively affecting cardiovascular, respiratory, musculoskeletal, and neuromuscular systems. These restrictions can lead to cellular changes that contribute to exercise intolerance. It has been confirmed that exercise intolerance is not always reversed with adequate hormonal therapy [33]. However, there is a significant group of patients who experience limitations in their ability to participate in daily sports activities, and this worsens their quality of life [33]. Regarding the musculoskeletal system, Guerin et al. surveyed women with hypothyroidism and concluded that hypothyroid women suffer more from muscle symptoms during exercise and at rest [34]. Hypothyroid patients with autoimmune thyroiditis were less likely to achieve moderate-intensity physical activity in comparison with healthy individuals [35]. Hypothyroid patients in thyroid replacement therapy are also less likely to comply with exercise [36]. Finally, patients with subclinical hypothyroidism had lower physical activity levels compared to healthy ones [37]. At present, there is limited knowledge about exercise tolerance in patients who have been treated for hypothyroidism, and there is not enough quantitative research on the effects of an interventional program. Therefore, additional research is required to explain this phenomenon.

Quality of life, physical activity, and thyroid dysfunction

Quality of life related to physical activity of patients with thyroid dysfunction has been also investigated and in a study on self-esteem, fatigue, quality of life of patients with thyroid disease, it has been found that engaging in moderate-intensity physical activity can improve the self-esteem levels, reduce fatigue, and enhance the quality of life of patients suffering from thyroid disease [38]. A randomized-controlled trial in women with subclinical hypothyroidism concluded that aerobic exercise could have outstanding results in health-related quality of life [39].

Animal studies

Experimental studies in animals have also been conducted to convey results about thyroid dysfunction and its impact on physical activity at the molecular level.

According to a study executed by Park et al. in 2016, exercise can help alleviate the symptoms of lethargy and reduce spatial learning ability caused by hypothyroidism. The research found that anaerobic exercise was more effective than aerobic exercise in restoring the function of the thyroid gland [40].

Body temperature and metabolic profile have gained interest regarding thyroid dysfunction impact on physical activity. Thyroidectomy has a specific impact on mechanisms that are involved in heat production more than muscular exercise. The increased catecholamine response in thyroidectomized dogs leads to a reduction in heat production. Furthermore, the metabolic effects of catecholamines were found to be relatively well preserved in thyroidectomized dogs. However, these findings cannot fully account for the effects of thyroidectomy [41]. A study on dogs to determine the effects of thyroid hormones on body temperature during exercise found that dogs who had undergone a thyroidectomy experienced a lower increase in temperature compared to preoperatively. On the other hand, dogs treated with thyroid hormones showed a greater increase in temperature. These findings suggest that there is an optimum level of thyroid hormones necessary to produce the typical increase in body temperature during physical activity. Having too much of these hormones can lead to exercise hyperthermia, while their deficiency can cause a reduction in temperature increase [42]. During a detailed study on the metabolic process, Paul et al carried out a study on the metabolism of free fatty acids (FFAs) in normal dogs and dogs that had undergone thyroidectomy. The study examined the effects of rest and acute exposure to cold on the dogs' FFA levels. FFA is an important energy source for the body, and its plasma concentration increases during periods of stress, such as fasting, acute cold exposure, and exercise. The study found that both normal and thyroidectomized dogs increased their FFA levels to the same extent during cold exposure. However, the FFA increase was significantly lower in thyroidectomized dogs. During cold exposure, the metabolic shift towards plasma FFA utilization was greater in thyroidectomized dogs than in normal dogs. Despite this, both groups utilized FFA at the same rate when expending the same amount of energy. The researchers concluded that the thyroid hormone does not directly control plasma FFA oxidation, but it is dependent on the metabolic rate of the tissue using it. Conversely, the absence of the thyroid hormone resulted in the suppression of FFA utilization. This study provides valuable insights into the role of FFA in energy metabolism during cold exposure and highlights the importance of thyroid function in FFA mobilization [43].

A study conducted in 2018 by Teixeira et al. compared the beneficial effects of hormonal treatment with those of aerobic exercise training in Wistar rats who had experienced myocardial infarction (MI). The results of the study showed that for rats who were intolerant to aerobic exercise, the benefits of T3 and T4 hormonal treatments were similar and, in some cases, even better than those of aerobic exercise. Therefore, the study suggests that low doses of thyroid hormones could be a more suitable alternative as adjunctive therapy after MI for patients who are intolerant to aerobic exercise [44]. On the other hand, a study by Kasimay Çakir et al. in 2017 on the effect of moderate or high-intensity exercise on hypothyroid rats exposed to acute stress shows that hypothyroid state could be protective in stress- and exhaustive exercise- induced oxidative damage [45].

Shin et al. studied the effect of treadmill exercise on memory and spatial learning ability in hypothyroid rats. Hypothyroid rats that exercised had better outcomes compared to hypothyroid rats without exercise [46].

## Conclusions

Thyroid function disorders indirectly affect physical activity through changes in the cardiovascular, metabolic, neuromuscular, and musculoskeletal systems. Hyperthyroid and hypothyroid patients can endure different programs of physical activity, while exercise has an impact on thyroid hormone production. Aerobic and anaerobic exercises are distinctly recommended among hyper- and hypothyroid patients. Further research is necessary to establish the optimal type and intensity of physical activity that is appropriate for each case, given the lack of comprehensive and reliable research data in this area.
